# Intraoperative Diagnosis and Surgical Management of Encapsulating Peritoneal Sclerosis: A Case Report

**DOI:** 10.7759/cureus.64515

**Published:** 2024-07-14

**Authors:** Shannon C South, Avani P Shah, Joseph R Hartigan

**Affiliations:** 1 Department of Medicine, Lake Erie College of Osteopathic Medicine, Bradenton, USA; 2 General Surgery, Ascension Health/St. Vincent's, Jacksonville, USA

**Keywords:** small bowel obstruction, peritoneal dialysis, abdominal cocoon, sclerosing encapsulating peritonitis, encapsulating peritoneal sclerosis

## Abstract

Encapsulating peritoneal sclerosis (EPS) is a serious complication of chronic peritoneal dialysis (PD) that results in encapsulation of the bowel in a thick, fibrocollagenous membrane. Given its rare and complex nature, diagnosis of EPS often arises late in the disease process or intraoperatively. We report the case of an 86-year-old male with a history of renal failure managed with PD who presented with multiple hospital admissions for recurrent abdominal pain and symptoms of small bowel obstruction. Open laparotomy revealed encasement of the entire abdominal cavity in a cocoon-like membrane, consistent with EPS, which was successfully managed with extensive excision and adhesiolysis. This discussion, enriched by unique radiographic insights and delineation of a surgical strategy, seeks to enhance the understanding of this underreported disease characterized by a lack of definitive treatment and an enigmatic pathophysiology.

## Introduction

Encapsulating peritoneal sclerosis (EPS), also known as abdominal cocoon syndrome, represents a rare and clinically challenging pathology characterized by abnormal peritoneal thickening and encapsulation [1]. This condition causes the development of fibrous membranes that surround abdominal organs, restricting their mobility and functionality [2]. The precise etiology of EPS remains elusive, although it has been categorized into idiopathic and secondary forms [3]. Secondary EPS arises in conjunction with various predisposing risk factors, most notably peritoneal dialysis (PD), which stands as the leading causative factor, alongside abdominal tuberculosis, a history of abdominal surgeries, and autoimmune disorders such as sarcoidosis [4]. Due to a lack of universal screening for EPS, there is marked variability in the incidence reported across studies. However, the one common finding among reports is an increased risk of developing EPS with a longer duration of PD [1,2,5,6].

Symptoms associated with EPS are diverse but frequently encompass abdominal pain, distension, nausea, vomiting, altered bowel habits, and recurrent episodes of both incomplete and complete small bowel obstruction [7]. These manifestations, while characteristic, lack specificity, rendering a preoperative diagnosis challenging and susceptible to misidentification as alternative gastrointestinal disorders [8].

The diagnosis of EPS requires a comprehensive medical history review and a heightened clinical index of suspicion, particularly in cases where patients present with recurrent bowel obstruction and signs indicative of peritonitis [9]. A crucial diagnostic tool is medical imaging, with CT scans being the preferred choice due to their capacity to provide a noninvasive confirmation of EPS [10]. In many cases, EPS is incidentally discovered during surgery (laparotomy) for other abdominal issues due to its nonspecific symptoms [2].

While early management can be done conservatively, the consensus among the current reports of EPS is that surgical management is almost always required [9,11,12]. In cases of EPS secondary to patients on PD, cessation of PD is recommended with supplementation with nutritional support [13]. Surgical management is typically also needed due to intestinal obstruction [2]. Here we report a case of an 86-year-old man diagnosed intraoperatively with EPS and managed successfully with surgical excision.

## Case presentation

An 86-year-old man presented to the emergency department with a two-week history of mild, generalized abdominal pain as well as associated nausea, vomiting, and weight loss. Prior, he had been admitted to the hospital numerous times for recurrent abdominal pain without significant findings following clinical evaluation. Past medical history included hypertension, coronary artery disease, peripheral vascular disease, aortic valvular stenosis, myelodysplasia, hypothyroidism, obstructive lung disease, atrial fibrillation, and chronic renal insufficiency. More specifically, the patient had stage four chronic kidney disease managed with hemodialysis three times a week, with a prior history of receiving PD. Past surgical history included abdominal aortic aneurysm repair, carotid endarterectomy, aortic valve replacement, appendectomy, triple hernia repair, and Watchman procedure. 

Computed tomography (CT) revealed dilated loops of the proximal small bowel and a transition point within the anterior abdomen attributed to adhesions. It was suspected that the patient had small bowel obstruction. At that point, there was no indication for immediate surgical intervention, and the patient was managed with analgesics, fluids, and bowel rest. On day two following admission, the patient experienced an episode of emesis overnight. A subsequent x-ray of the small bowel with Gastrografin showed prominent duodenal dilatation; however, the patient was unable to tolerate adequate oral contrast for further examination. Having exhausted the remaining conservative approaches, diagnostic laparoscopy was determined to be the best next step in care. 

An attempted laparoscopic approach was made; however, the patient's intolerance to pneumoperitoneum, evidenced by hemodynamic instability, necessitated a transition to an open procedure. A midline incision was made, and soft tissue was dissected down to the fascia, which was then incised. A thick, white, rind-like membrane encased the entire abdominal cavity, obscuring the visibility of intra-abdominal organs and structures. Tedious excision of this fibrous tissue with adhesiolysis was performed, beginning at the liver edge and working downward. A transition point causing partial obstruction was identified at the jejunum, indicated by a fairly dilated small bowel. Adhesions from the proximal jejunum to the right colon were excised. A peanut sponge was used for careful dissection. Irrigation with warm saline and inspection for hemostasis followed. Post-operatively, the patient was started on total parenteral nutrition to maintain adequate nutrition and prevent further weight loss. No significant complications were encountered throughout the patient's recovery. During outpatient follow-up in the clinic post-procedure, the patient reported feeling markedly better, noting a significant reduction in abdominal pain. 

**Figure 1:**
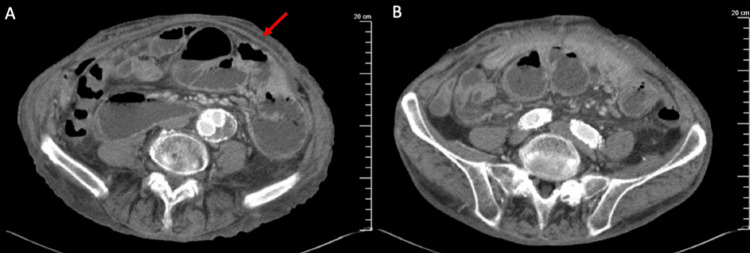
Preoperative abdominal CT (A) Peritoneal thickening (red arrow); (B) thickened subfascial plane CT, computed tomography

A retrospective review of the preoperative CT revealed characteristic radiographic features of EPS, particularly peritoneal thickening (Figure 1A), as well as a thickened subfascial plane (Figure 1B).

## Discussion

EPS is a clinically challenging pathology that results in peritoneal thickening and encapsulation in a fibrocollagenous membrane [2]. Characterized by vague symptoms, including abdominal pain, nausea, and vomiting, EPS often resembles alternative gastrointestinal ailments [8]. Due to the challenges of diagnosing EPS based solely on clinical presentation, diagnosis relies heavily on CT imaging. CT stands out as the most thoroughly researched imaging method for diagnosing EPS and is the only modality in which information on sensitivity and specificity is available. It has been identified as an effective tool for diagnosing EPS. Characteristic findings include fluid loculation, diffuse inflammatory fibrotic changes, adherent bowel loops, as well as peritoneal enhancement, thickening, and calcification [14]. Even so, EPS poses a diagnostic dilemma for radiologists since it can be easily overlooked on CT, as illustrated in our case, necessitating a keen clinical suspicion for prompt identification. 

Given the association of EPS with various conditions such as prior abdominal surgery and PD, it is crucial to maintain heightened awareness when assessing at-risk populations. This is especially important given the substantial morbidity of EPS, which has been reported to be as high as 50% [15]. In this particular case, the patient’s medical history involved a longstanding history of receiving PD for renal failure, along with considerable abdominal surgery, including abdominal aortic aneurysm repair, appendectomy, and triple hernia repair. These factors likely compounded his risk of developing EPS. 

Another noteworthy observation is that EPS has been reported to develop or worsen following cessation of PD therapy [15]. The patient presented with a pre-existing history of PD treatment, however, had subsequently transitioned to hemodialysis three times weekly. This finding underscores the necessity for in-depth research into the temporal relationship between dialysis treatment modification and disease progression. A potential avenue for further study includes screening patients intending to alter their current PD regimen using CT imaging to evaluate for EPS. Of note, one significant limitation of this study arises from the uncertainty surrounding the duration of the patient's prior PD therapy.

It is crucial to recognize that EPS secondary to PD can closely resemble tuberculous peritonitis. Despite similarities, distinguishing features exist. Pathological examination of EPS typically shows loss of the mesothelial cell layer, fibrin deposition, fibroblast swelling, and infiltration of mononuclear cells. In contrast, tuberculous peritonitis exhibits a granulomatous inflammatory response characterized by epithelioid macrophages, Langerhans giant cells, and central caseous necrosis. Another distinctive feature of tuberculous peritonitis is the calcification of mesenteric and retroperitoneal lymph nodes, which can be identified through radiographic evaluation. Moreover, cloudy ascites and fever are commonly observed in patients with abdominal tuberculosis, symptoms that were notably absent in our patient [16]. 

Numerous studies have documented favorable outcomes following surgical intervention for EPS. In a retrospective observational study conducted by Kawanishi et al. in 2019, the survival rates at one, two, and three years after surgical intervention were 91%, 83%, and 77%, respectively [12]. Similarly, Kawanishi et al. in 2006 demonstrated that 81 out of 86 patients who underwent surgical treatment experienced significant symptomatic improvement [17]. In our case, detailed excision of the fibrotic membrane during laparotomy led to successful symptom resolution, serving as another compelling piece of evidence supporting favorable outcomes associated with the surgical management of EPS. However, we recognize that EPS is likely a highly recurrent disease, even following surgical intervention. It has been hypothesized that recurrent disease represents an irreversible pathologic condition due to peritoneal microvascular hyperplasia [17]. For this reason, future research endeavors should prioritize investigating factors contributing to recurrence and exploring therapeutic options for symptoms that persist or recur following surgical excision.

## Conclusions

In conclusion, EPS was diagnosed through direct operative visualization of peritoneal encapsulation, supplemented by retrospective identification of characteristic features on CT. This demonstrates that close collaboration between radiologists and surgeons is critical, utilizing both imaging modalities and surgical findings to ensure accurate diagnosis and optimal patient care. This case highlights the importance of considering EPS as a cause of intestinal obstruction, particularly for high-risk patients with a longstanding history of receiving PD. Moreover, it illustrates that when discovered intraoperatively, EPS may be managed with meticulous excision of the fibrocollagenous membrane. By identifying and excising the fibrous tissue causing intestinal adhesion, resolution of patient symptoms may be achieved. Additionally, the radiographic imaging presented in this discussion contributes to the existing literature, while the outlined surgical management plan has proven successful. Although recognition of EPS has increased over the last decade, it remains a challenging disease in which further investigations are warranted to both deepen our understanding and enhance patient outcomes.

## References

[1] Moinuddin Z, Summers A, Van Dellen D, Augustine T, Herrick SE (2014). Encapsulating peritoneal sclerosis-a rare but devastating peritoneal disease. Front Physiol.

[2] Tannoury JN, Abboud BN (2012). Idiopathic sclerosing encapsulating peritonitis: abdominal cocoon. World J Gastroenterol.

[3] Basara Akin I, Altay C, Celik A, Secil M (2019). Computed tomography features of encapsulating peritoneal sclerosis. Can Assoc Radiol J.

[4] Fujiwara S, Akaishi R, Yokosawa T (2023). Sclerosing encapsulating peritonitis: abdominal cocoon. Cureus.

[5] Petrie MC, Traynor JP, Mactier RA (2016). Incidence and outcome of encapsulating peritoneal sclerosis. Clin Kidney J.

[6] Nitsch D, Davenport A (2015). Designing epidemiology studies to determine the incidence and prevalence of encapsulating peritoneal sclerosis (EPS). Perit Dial Int.

[7] Gayomali C, Hussein U, Cameron SF, Protopapas Z, Finkelstein FO (2011). Incidence of encapsulating peritoneal sclerosis: a single-center experience with long-term peritoneal dialysis in the United States. Perit Dial Int.

[8] Akbulut S (2015). Accurate definition and management of idiopathic sclerosing encapsulating peritonitis. World J Gastroenterol.

[9] Machado NO (2016). Sclerosing encapsulating peritonitis: review. Sultan Qaboos Univ Med J.

[10] Singhal M, Krishna S, Lal A (2019). Encapsulating peritoneal sclerosis: the abdominal cocoon. Radiographics.

[11] Danford CJ, Lin SC, Smith MP, Wolf JL (2018). Encapsulating peritoneal sclerosis. World J Gastroenterol.

[12] Kawanishi H, Banshodani M, Yamashita M, Shintaku S, Dohi K (2019). Surgical treatment for encapsulating peritoneal sclerosis: 24 years’ experience. Perit Dial Int.

[13] Jagirdar RM, Bozikas A, Zarogiannis SG, Bartosova M, Schmitt CP, Liakopoulos V (2019). Encapsulating peritoneal sclerosis: Pathophysiology and current treatment options. Int J Mol Sci.

[14] Vlijm A, van Schuppen J, Lamers AB, Struijk DG, Krediet RT (2011). Imaging in encapsulating peritoneal sclerosis. NDT Plus.

[15] Brown EA, Bargman J, van Biesen W (2017). Length of time on peritoneal dialysis and encapsulating peritoneal sclerosis - position paper for ISPD: 2017 update. Perit Dial Int.

[16] Tseng WC, Tarng DC (2012). Cocoon-like fibroadhesive tuberculous peritonitis in a peritoneal dialysis patient. Chin J Physiol Sci.

[17] Kawanishi H, Moriishi M, Tsuchiya S (2006). Experience of 100 surgical cases of encapsulating peritoneal sclerosis: investigation of recurrent cases after surgery. Adv Perit Dial.

